# Bemotrizinol and the modernization of US sunscreen regulation

**DOI:** 10.1016/j.jdin.2026.06.003

**Published:** 2026-06-16

**Authors:** Umayr R. Shaikh, Christopher G. Richter, Leela K. Raj, Afrin Mirza, Harold William Higgins, Jane M. Grant-Kels

**Affiliations:** a Department of Dermatology, University of Pennsylvania, Philadelphia, Pennsylvania; b Georgetown University School of Medicine, Washington, DC; c University of Texas Medical Branch John Sealy School of Medicine, Galveston, Texas; d Sidney Kimmel Medical College at Thomas Jefferson University, Philadelphia, Pennsylvania; e Department of Dermatology, University of Connecticut, Farmington, Connecticut; f Department of Dermatology, University of Florida Health, Gainesville, Florida

**Keywords:** bemotrizinol, FDA, over the counter, photoprotection, sunscreens, ultraviolet light filter, zinc oxide

*To the Editor:* On June 9, 2026, the US Food and Drug Administration (FDA) finalized an administrative order adding bemotrizinol to the over-the-counter sunscreen monograph as a generally recognized as safe and effective (GRASE) active ingredient at concentrations of up to 6% for adults and children aged 6 months and older.^1^ It is the first new UV filter added to the monograph since 1999.^1^^,^^2^ The approval has been framed as a photoprotection breakthrough, but its durable significance may be procedural: it is the first sunscreen approval to emerge from a regulatory architecture rebuilt specifically to prevent such delays.^2^

Bemotrizinol, marketed abroad as Tinosorb S and Parsol Shield, has more than 2 decades of use across Europe, Australia, and Asia.^3^^,^^4^ It offers broad-spectrum coverage with limited systemic absorption, and its defining advantage is photostability.^1^^,^^2^^,^^4^ Unlike avobenzone, the principal nonmineral UV-A filter used in US sunscreens, bemotrizinol remains stable after UV exposure.^4^ Combined with zinc oxide, it can provide broad-spectrum protection with less white cast.^5^ This offers a meaningful advantage because mineral sunscreen residue remains a barrier to use among patients with darker skin tones.^5^

The question is not why bemotrizinol was approved, but why a filter with this record took so long to reach US patients. The answer is regulatory rather than scientific. Because the FDA regulates sunscreens as over-the-counter drugs, UV filters fall under the monograph system, which for decades relied on slow notice-and-comment rulemaking.^2^^,^^3^ The Sunscreen Innovation Act of 2014 created an expedited pathway but yielded no approvals, in part because the FDA required testing such as Maximum Usage Trials that was not harmonized with international standards.^1^^,^^2^ Eight filters submitted under the act remain unapproved.^3^

What changed, however, is easy to miss. The Coronavirus Aid, Relief, and Economic Security Act of 2020 replaced rulemaking with a user fee–funded administrative order process while preserving evidentiary standards.^1^^,^^2^ Bemotrizinol was reviewed through this new framework and ultimately approved in June 2026.^1^ The machinery for timely approvals now exists, and bemotrizinol provides the first tangible evidence that rigorous GRASE review and regulatory efficiency are not inherently in conflict.^1^^,^^2^

The clinical stakes of closing this gap are meaningful but should not be overstated. Sunscreen adherence is strongly influenced by cosmetic acceptability.^5^ Greasy, unstable, or residue-leaving formulations are used less consistently.^5^ More elegant and photostable products may narrow the gap between demonstrated efficacy and everyday effectiveness, particularly among patients underserved by mineral-only options.^4^^,^^5^ No single filter will alter skin cancer incidence on its own. The likely benefit is behavioral, as better-tolerated products are used more consistently.^5^

Bemotrizinol’s arrival is worth celebrating, but the milestone is not this ingredient itself. Rather, it is whether the FDA will use its streamlined authority to evaluate filters still under review while incorporating the global postmarketing evidence available for many of them.^2^^,^^3^ Its success should be measured not by the approval of a single molecule but by whether 2026 marks the beginning of a more globally aligned approach to sunscreen regulation, or merely an exception that proves how rare such progress remains.^2^^,^^3^

## Conflicts of interest

None disclosed.
